# Actinobacteria: An eco-friendly and promising technology for the bioaugmentation of contaminants

**DOI:** 10.1016/j.btre.2021.e00679

**Published:** 2021-10-05

**Authors:** Christina-Injan Mawang, Adzzie-Shazleen Azman, Aalina-Sakiinah Mohd Fuad, Mariana Ahamad

**Affiliations:** a Acarology Unit, Infectious Disease Research Centre, Institute for Medical Research, Ministry of Health Malaysia, National Institutes of Health Complex, Setia Alam, Shah Alam, Selangor, 40170, Malaysia; b School of Science, Monash University Malaysia, Jalan Lagoon Selatan, Bandar Sunway, Selangor, 47500, Malaysia; c Department of Biomedical Science, Kulliyyah of Allied Health Sciences, International Islamic University Malaysia Kuantan Campus, Jalan Sultan Ahmad Shah, Bandar Indera Mahkota, Kuantan, Pahang 25200, Malaysia

**Keywords:** Actinobacteria, Bioaugmentation, Heavy metals, Pesticides

## Abstract

Over the past two decades, various eco-friendly approaches utilizing microbial species to clean up contaminated environments have surfaced. In this aspect, actinobacteria have demonstrated their potential in contaminant degradation. The members of actinobacteria phylum exhibits a cosmopolitan distribution, which means that they can be found widely in both aquatic and terrestrial ecosystems. Actinobacteria play important ecological roles in the environment, such as degrading complex polymers, recycling compounds, and producing bioactive molecules. Hence, using actinobacteria to clean up contaminants is an attractive method in the field of biotechnology. This can be achieved through the green technology of bioaugmentation, whereby the degradative capacity of contaminated areas can be greatly improved through the introduction of specific microorganisms. This review describes actinobacteria as an eco-friendly and a promising technology for the bioaugmentation of contaminants, with focus on pesticides and heavy metals.

## Highlights

• The concept and different approaches of bioaugmentation.
• Actinobacteria as a potential for cleaning up contaminants.
• Actinobacteria with pesticide degrading abilities and/or heavy metal resistance and removal capabilities.
• Studies on bioaugmentation of soils contaminated with different pesticides and heavy metals by actinobacteria.

## 1 Introduction

The ever-increasing anthropogenic activities around the world have resulted in several areas being severely contaminated with hazardous contaminants such as pesticides and heavy metals [1,2]. Pesticides are widely used to control pests and are possibly the most extensively distributed contaminants in the environment [3]. There are countless long-term contaminated sites with great levels of pesticides due to the dumping of obsolete pesticide stocks. Additionally, pollution caused by heavy metals is also among the top most relevant environmental problems today [4]. The extensive heavy metal usage in numerous practices has led to their global spread in silt, soil, waste, and wastewater. Toxic metals polluting the environment came about from several human activities, mainly industrial activities, although agriculture, municipal landfill, and sewage disposal activities had similarly contributed to the pollution [4,5]. Considering these problems, eco-friendly methods to clean up polluted environments by means of various microbial species have emerged to be the preferred pollutant clean-up approach. This approach is more acceptable as it is less intrusive and more remedial of soil functions in comparison to standard physicochemical approaches [6]. Moreover, biological approaches have better flexibility as they involve living systems, hence having the ability to perform complex reactions such as degrading organic pollutants and converting inorganic compounds into nontoxic products [7,8].

Contaminated areas naturally contain microorganisms having the ability to degrade an extensive range of contaminants. However, several pollutants exhibit resistance to biodegradation due to various factors such as low water solubility, low biodegradability, low bioavailability, high toxicity, and high stability [9]. Besides, certain compounds may be inefficiently used as substrates for microbial metabolism. Due to the complexity of the chemical structure of some compounds, a microbial consortium may be required for them to undergo biodegradation. Recalcitrant compounds (slowly biodegradable or non-biodegradable compounds) can be new in many cases, and therefore microorganisms are unlikely to be adapted in using them as substrates [10]. Bioaugmentation is a suitable approach to overcome these problems as its treatment can be modified to the targeted pollutant dominantly present in the environment. It can target not only pollutants in increasing amounts but also those present in high concentrations [9].

Amongst environmental microorganisms, actinobacteria have been broadly reported as probable bioremediation agents [3,11]. In nature, actinobacteria are dominant colonizers [12] and are important for recycling refractory biomaterials through humus formation and decomposition [13]. Due to their beneficial functions in nature, actinobacteria have received notable attention, especially on their capability to degrade organic and inorganic pollutants. In this review, a compilation of successful usage of actinobacteria in bioaugmentation within 15 years is described with many case studies. Although, there are many other contaminants present worldwide, this review focuses on pesticides and heavy metals as these two are the most prevalent contaminants in the environment.

## 2 General concept of bioaugmentation

In general, bioremediation refers to the method of using microorganisms, plants or their enzymes to reduce or remove environmental contaminants or toxic substances. The detoxification can be achieved through different methods including the addition of nutrient to the affected area (biostimulation or biorestoration) and the addition of microbe with specific characteristic (bioaugmentation) [14]. Therefore, it is important to understand the principal and differentiate all bioremediation methods to achieve the main goal; to reduce the concentration or level of toxic contaminants to acceptable level following the environmental requirement or to completely remove it [15].

**Fig. 1:**
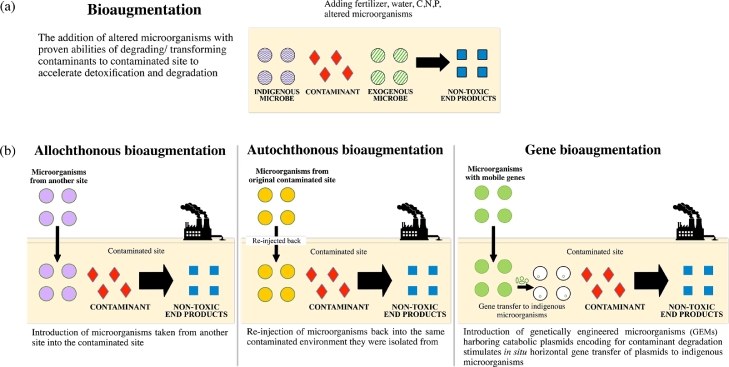
(a) Bioaugmentation method; (b) Three different approaches of bioaugmentation.

Bioaugmentation is a type of bioremediation method whereby environmental samples such as soil, sediments and water are inoculated with microorganisms characterized with the preferred catalytic abilities to accelerate the degradation of contaminants (Fig. 1a) [[16], [17], [18]]. It is mainly carried out in areas where there is low number of autochthonous microorganisms with the ability to degrade the contaminant, and/or the necessary catabolic pathways needed to metabolize pollutants is unavailable in the native population [16,17]. In selecting the appropriate strains for bioaugmentation, the following features of the microorganisms need to be considered: good synergistic interactions among introduced and indigenous microbes, high capacity to degrade contaminants, ease of cultivation, fast growth, endurance in high concentrations of pollutants, and survival in various environmental conditions [17,19].

Based on the origin of the inoculants, bioaugmentation has three different approaches (Fig. 1b). In allochthonous bioaugmentation, microorganisms taken from another site are introduced into the contaminated site, whereas in autochthonous bioaugmentation, microorganisms originally from the polluted site are re-injected back into the same polluted environment. In gene bioaugmentation, genetically engineered microorganisms (GEMs) equipped with genes encoding for enzymes responsible for specific desired functions are introduced directly into the environment. This is to enhance the rate of contaminant degradation by increasing, through horizontal gene transfer, the number and diversity of indigenous microorganisms to metabolize the target contaminants [[18], [20], [21], [22]]. Examples of plasmid that have been reported in plasmid-mediated bioaugmentation includes pJP4 for 2,4-dichlorophenoxyacetic acid (2,4-D) degradation [23], pDOC for chlorpyrifos degradation [24], and pDOD for dichlorodiphenyltrichloroethane (DDT) degradation [20]. Degradation of these pesticides are important as they are widely used in agriculture but have caused serious toxicological and environmental problems.

As such, there has been an increasing interest in using Actinobacteria to remove toxic substances and soil contaminants via bioaugmentation. Through metagenomic analysis done by various researchers, Actinobacteria is either the second or third major groups found in contaminated soils with heavy metals [25], [26], [27], and pesticides [28,29]. This proves that Actinobacteria is a good candidate to be used in bioaugmentation.

## 3 Actinobacteria

Actinobacteria is one of the largest phylum under Bacteria domain and can be found in a wide range of terrestrial and aquatic ecosystems. Actinobacteria are Gram-positive bacteria with > 50% of guanine and cytosine (*G* + *C*) content in their DNA. Generally, actinobacteria are recognized as filamentous bacteria due to their ability to form substrate mycelium and aerial mycelium [30]. Until 2016, 374 genera of actinobacteria have been recorded [31]. Actinobacteria can be found either as *Streptomyces*, which is the dominant genus in the group, or as non-*Streptomyces* actinomycetes such as the genus of *Actinomyces, Kitasatospora, Micromonospora, Nocardia, Micrococcus, Arthrobacter* and *Rhodococcus* [32]. *Streptomyces* covers a large number of species with great diversity in culture conditions [33]. In contrast, non-*Streptomyces* actinomycetes are strains with low frequency of isolation under normal conditions and most of them require specific isolation, preservation and cultivation methods [34,35].

The phylum Actinobacteria represents the most recognized group of microorganisms with the ability to produce bioactive compounds. Thus, actinobacteria have received great interest in various applications in pharmaceuticals, biotechnology, food industries, agriculture and in the enzyme industry [36], [37], [38]. Moreover, there is also interest to use Actinobacteria to assist in pollutant clean-up. Actinobacteria provides an economic and safe biological method for removal of contaminants as they can metabolize the contaminants for their growth [39].

## 4 Actinobacteria with pesticide degrading abilities

Over the years, there have been many research findings on pesticide degradation by actinobacteria. It has been noted that *Streptomyces* and non-*Streptomyces* actinomycetes of the genus *Arthrobacter, Frankia, Gordonia, Kocuria, Nocardioides* and *Rhodococcus* are among the commonly reported pesticide degrading actinobacteria. These microorganisms exhibit abilities to grow, metabolize and degrade several chemical families of pesticides, including organophosphorous (chlorpyrifos, dimethoate), organochlorine (endosulfan, lindane, pentachlorophenol, pentachloronitrobenzene, hexachlorobenzene), triazine (atrazine, simazine, terbuthylazine), synthetic pyrethroids (deltamethrin, cypermethrin) and benzimidazole (carbendazim) compounds [3,40]. Examples of actinobacteria with pesticide degrading abilities are listed in Table 1.

**Table 1:** Actinobacteria with pesticide degrading abilities.

Actinobacteria	Pesticide degraded	Source of isolation	Ref
*Arthrobacter* sp.	α-endosulfan, β-endosulfan	Soil from different agricultural fields contaminated with pesticides, India	[41]
*Arthrobacter* sp. strain DAT1	Atrazine	Atrazine-contaminated agricultural soil in Hebei Province, China	[42]
*Arthrobacter* sp. strain AK-YN10	Atrazine	Soil from atrazine-contaminated agricultural field in Maharashtra, India	[43, 44]
*Arthrobacter* sp. C3	Atrazine	Soil from atrazine-sprayed corn field in China	[45]
*Arthrobacter* sp. SD3–25	Simazine	Soil from simazine-contaminated farmland in Liaoning Province, China	[46]
*Arthrobacter aurescens* TC1	Terbuthylazine	Soil from atrazine-contaminated spill site in South Dakota, USA	[47, 48]
*Frankia alni* ACN14a	Atrazine	Not reported	[49]
*Frankia* sp. EuI1c	Atrazine	Not reported	[49]
*Gordonia* sp. JAAS1	Chlorpyrifos	Paddy field soil exposed to continuous chlorpyrifos applications in Tamil Nadu, India	[50]
*Kocuria* sp. CL2	Pentachlorophenol	Sludge of pulp and paper mill	[51]
*Kocuria* sp. DAB-1Y	Lindane	Soil from a contaminated site in Lucknow, India with history of lindane application	[52]
*Kocuria kristinae*	Chlorpyrifos	Chlorpyrifos-contaminated agricultural soil in India	[53]
*Kocuria turfanensis*	Dimethoate	Soil from long history of pesticide application in India	[54]
*Nocardioides* sp. strain PD653	Pentachloronitrobenzene, Hexachlorobenzene, Pentachlorophenol	Soil from an agricultural field contaminated with pentachloronitrobenzene in Japan	[55]
*Nocardioides* sp. strain SG-4G	Carbendazim	Carbendazim-exposed soil in Australia	[56]
*Nocardioides soli*	Carbendazim	Soil under long-term applications of carbendazim in Changzhou, China	[57]
*Rhodococcus* sp. BCH2	Atrazine	Soil from grape farm with long-term atrazine treatment near Sangli, India	[58]
*Rhodococcus* sp. strain MB-P1	Atrazine	Atrazine-contaminated soil in India	[59]
*Rhodococcus* sp. djl-6	Carbendazim	Carbendazim-treated soil of a vegetable field in Taixing, China	[60]
*Rhodococcus erythropolis* djl-11	Carbendazim	Soil from vineyards in Rhizao, China with 10-year history of carbendazim application	[61]
*Rhodococcus jialiangiae* djl-6–2	Carbendazim	Sludge of a carbendazim wastewater treatment facility in Jiangsu province, China	[62]
*Rhodococcus qingshengii* djl-6	Carbendazim	Carbendazim-contaminated soil from a vegetable field in Jiangsu province, China	[63]
*Streptomyces aureus* HP-S-01	Deltamethrin, Cypermethrin	Activated sludge from a pyrethroid manufacturing wastewater treatment system in Zhongshan, China	[64, 65]
*Streptomyces* sp. M7	α-HCH, β-HCH, Lindane (γ-HCH)	Wastewater sediment of a copper filter plant in Tucuman, Argentina	[66, 67, 68]
*Streptomyces* sp. strains A2, A5, A11	Lindane	Organochlorine pesticide-contaminated soil in Santiago del Estero, Argentina	[69]

## 5 Bioaugmentation of pesticide-contaminated soils using pesticide degrading actinobacteria

Although various studies have been conducted to isolate and characterize the degrading capacity of pesticide degrading actinobacteria, and with some studies showing bioremediation potentials [45, 51, 56, 70, 68], not all pesticide degrading actinobacteria have been studied further on their potentials to be used for the bioaugmentation of pesticide-contaminated soils. Table 2 describes and summarizes studies that have reported on the use of pesticide degrading actinobacteria to decontaminate pesticides present in soil via bioaugmentation.

**Table 2:** Characteristics of pesticide degrading actinobacteria used in the bioaugmentation of pesticide contaminated soils.

Actinobacteria	Pesticide degraded	Dosage (mg/kg)	Inoculum size	Removal efficiency/ comments	Ref
*Arthrobacter* sp.	α-endosulfan, β-endosulfan	50	2 × 10^8^ CFU/g	Degradation of 73% α-endosulfan and 75% β-endosulfan after 6 weeks	[41]
*Arthrobacter* sp. strain DAT1	Atrazine	400	1.3 × 10^7^ CFU/g	>95% of atrazine removal in 3 days	[73]
*Arthrobacter* sp. strain AK-YN10	Atrazine	100	2.5 × 10^7^ CFU per 250 g	Complete removal of atrazine within 12 days	[44]
*Arthrobacter* sp. SD3–25	Simazine	200	3.3 × 10^6^ CFU/g	>90% of simazine removal within 7 days	[46]
*Arthrobacter aurescens* TC1	Terbuthylazine	3.8	5 × 10^7^ - 2 × 10^8^ CFU/g	95% of terbuthylazine removal within 3 days	[48]
*Rhodococcus jialiangiae* djl-6–2	Carbendazim	10	1 × 10^8^ CFU/g	Almost 100% carbendazim removal after 20 days	[77]
*Rhodococcus qingshengii* djl-6	Carbendazim	2, 8	1 × 10^8^ CFU/g	> 93% of carbendazim removal after 14 days of incubation	[76]
*Streptomyces aureus* HP-S-01	Cypermethrin, 3-PBA	50	1 × 10^6^ CFU/g	▪ Removal of 81.1% cypermethrin and 73.5% 3-PBA after 10 days (field experiments) ▪ Degradation of other pyrethroids such as bifenthrin, cyfluthrin, fenvalerate, fenpropathrin and permethrin	[64, 65]
*Streptomyces* consortium (strains A2, A5, A11, M7)	Lindane	35	1 × 10^7^ CFU/g	▪ *Streptomyces* consortium immobilized in cloth sachet ▪ Lindane removal from concentrated slurry (soil: water ratio of 2:3) within 7 days	[79]
*Streptomyces* consortium (strains A2, A5, A11, M7)	Lindane	100	1 × 10^8^ CFU/g	▪ 81–87% of lindane removal in bioaugmented biomixtures at 60 days of incubation	[80]
*Streptomyces* consortium (strains A2, A5, A11, M7)	Lindane	2	2 g/kg, wet weight	▪ Microcosms studies: 61–86% of lindane removal in bioaugmented and biostimulated soils after 14 days ▪ Mesocosms studies: 68–82% of lindane removal in bioaugmented and biostimulated soils after 63 days	[81, 82]

Endosulfan is an insecticide that has been used extensively worldwide to control pests on crops. It is a mixture of two stereoisomers, α- endosulfan and β-endosulfan, in a ratio of 7:3. Endosulfan is toxic, bioaccumulative and persistent in the environment [41,71]. Kumar et al. (2008) reported on the bioaugmentation of endosulfan-contaminated soil with *Arthrobacter* sp. [41]. Through soil microcosm studies, inoculation of *Arthrobacter* sp. (2 × 10^8^ CFU/g) into endosulfan-contaminated soil resulted in 73% and 75% degradation of α- and β-endosulfan (50 mg/kg), respectively after 6 weeks. As for the uninoculated soils, degradation of α- and β-endosulfan after 6 weeks was 26% and 23%, respectively [41].

Atrazine is one of the most widely used and toxic herbicides to control weeds in crops. It is a pollutant of great concern due to its high potential to cause soil and water contamination [42,72]. The genus *Arthrobacter* has been recognised as capable of degrading many types of xenobiotics [3]. An atrazine degrading *Arthrobacter* sp. strain DAT1 was reported to be able to utilize atrazine as a sole nitrogen source for growth and harboured atrazine-degrading genes (*atzB, atzC* and *trzN*) [42]. Wang et al. (2013) later investigated the potential of strain DAT1 in remediating heavily atrazine-contaminated soil [73]. Strain DAT1 demonstrated highly effective removal of atrazine (400 mg/kg) from contaminated soil, whereby the inoculation of strain DAT1 (1.3 × 10^7^ CFU/g) into both autoclaved and non-autoclaved soils had resulted in a nearly complete removal of atrazine (>95%) in 3 days. Moreover, results on the inoculants durability/ survival and catabolic potential for soil remediation using terminal restriction fragment length polymorphism (TRFLP) and quantitative PCR (qPCR) targeting the atrazine-degrading genes demonstrated a steady increase of the tested genes and the relative abundance of strain DAT1 in the bioaugmented soil throughout the bioremediation experiment [73].

In another study, Sagarkar et al. (2014) conducted soil mesocosm studies and demonstrated that bioaugmentation with an atrazine degrading microbial consortium (which also includes *Arthrobacter* sp. strain AK-YN10) showed the best removal strategy, with 90% of atrazine degradation in 6 days for soil with previous atrazine exposure [43]. However, removal of atrazine from soil without history of atrazine exposure needed 15 days. Moreover, length heterogeneity PCR (LH-PCR) monitoring of the bacterial community profile in all mesocosms indicated that the natural microflora in soil was not affected by the bioaugmentation process. This study showed that efficient bioaugmentation of atrazine using a microbial consortium could be successfully scaled up into pilot scale treatments [43]. Sagarkar et al. (2016) studied the bioaugmentation potential of *Arthrobacter* sp. strain AK-YN10 on atrazine contaminated soil by microcosm experiments [44]. Strain AK-YN10 demonstrated 99% of atrazine degradation within 30 h when tested on media supplemented with 1000 mg/L of atrazine. Moreover, results from microcosm experiments with 100 mg/kg of atrazine showed complete removal of atrazine within 12 days in soil bioaugmented with strain AK-YN10 (2.5 × 10^7^ CFU per 250 g soil). Strain AK-YN10 also demonstrated the capacity to degrade other triazine pesticides, including simazine, terbuthylazine, ametron, prometron, ametryn and prometryn, indicating that it could be a good candidate for bioremediation of soils contaminated by triazine pesticides [44].

Simazine is also another widely used herbicide to control weeds. The presence of simazine in the environment is of concern due to the persistence and toxicity of simazine [46]. Guo et al. (2014) had investigated the degradation ability of a simazine-degrading *Arthrobacter* sp. SD3–25. It was found that when comparing between soil without inoculation and soil inoculated with strain SD3–25, >90% of simazine (200 mg/kg) in the inoculated soil was removed within 7 days. Moreover, sucrose amendment in the bioaugmented soil had increased the growth of strain SD3–25, and this was observed through the increased proportion of *atzB, atzC* and *trzN* genes. This had led to enhanced simazine biodegradation in the bioaugmented soil [46].

Another type of herbicide, terbuthylazine had displaced atrazine in most EU countries. It has been increasingly used to control weeds and have been frequently detected in the environment, making it an emerging concern [48,74]. The soil bacterium *Arthrobacter aurescens* TC1 was reported to be capable of utilizing atrazine and other chloro‑s-triazine herbicides including terbuthylazine as nitrogen and/or carbon sources [47,48]. Silva et al. (2015) demonstrated successful application of *Arthrobacter aurescens* TC1 to eliminate terbuthylazine from freshly spiked or 4-month aged soils [48]. Inoculation of strain TC1 (5 × 10^7^ and 2 × 10^8^ cfu/g of soil) in freshly spiked soil resulted to rapid elimination of about 95% of initial dose of terbuthylazine (3.8 mg/kg) within 3 days. In contrast, about 70% of terbuthylazine applied remained in the non-bioaugmented soil until the final day of the 14-days experiment. Comparatively, terbuthylazine aging in soil led to a decreased rate and extent of terbuthylazine biodegradation, particularly in bioaugmented soils (inoculum density of 8 × 10^7^ CFU/g of soil). Nonetheless, the use of a 10-fold higher inoculum density will be able to ensure an absolute elimination of terbuthylazine. This showed the importance of inoculum density of the bioaugmentation bacterium in influencing the time needed to achieve effective soil bioremediation [48].

The extensive use of the fungicide carbendazim to control fungal diseases in agriculture had led to its accumulation and environmental contamination worldwide. Furthermore, carbendazim undergoes slow degradation and can persist in bare soil for a long time [75,76]. The genus *Rhodococcus* can utilize recalcitrant and toxic compounds for its growth and metabolism, and majority of carbendazim-degrading bacteria belongs to this genus [75]. The carbendazim-degrading *Rhodococcus jialiangiae* djl-6–2 was reported to be capable of utilizing carbendazim as the sole carbon and nitrogen source for growth [62,77]. Wang et al. (2010) had conducted a pilot scale bioremediation study and reported on the successful bioaugmentation of carbendazim in soil using strain djl-6–2. Inoculation of strain djl-6–2 (1 × 10^8^ CFU/g) had promoted the degradation of carbendazim (10 mg/kg) in both sterilized soil and non-sterilized soil, with the degradation rate of carbendazim in non-sterilized soil being higher than sterilized soil during the first 6 days. No differences were observed between the final degrading rates in both soils after 20 days, indicating the ability of strain djl-6–2 to cooperate well with indigenous microorganisms in the soils and exercising its bioaugmentation function [77].

In another study, Chuang et al. (2021) reported on the potential effects of another carbendazim-degrading strain, *Rhodococcus qingshengii* djl-6 in the bioaugmentation of carbendazim contaminated soil [76]. The inoculation of strain djl-6 (1 × 10^8^ CFU/g) had significantly changed the response pattern of soil microbial communities to carbendazim. Moreover, strain djl-6 had enhanced the influence of carbendazim on bacterial community composition and reduced the fungicidal activity of carbendazim. Under strain-inoculated conditions, more than 93% of carbendazim was removed from both low dose (2 mg/kg) and high dose (8 mg/kg) carbendazim-treated soils after 14 days of incubation, as compared to 29% of carbendazim removal from uninoculated soils [76].

Synthetic pyrethroids insecticides are used to control insect pests in both agriculture and home. Although potently neurotoxic against insects and low toxicities for mammals, it is potentially harmful to human health and the environment [64,65]. The deltamethrin-degrading *Streptomyces aureus* HP-S-01 was reported to be highly effective in degrading pyrethroids such as deltamethrin, bifenthrin, cyfluthrin, cypermethrin, fenvalerate, fenpropathrin and permethrin, and also their common metabolite, 3-phenoxybenzaldehyde (3-PBA) in liquid cultures [64,65]. Chen et al. (2012) had conducted laboratory and field-scale experiment to investigate the ability of inoculated strain HP-S-01 to eliminate β-cypermethrin and 3-PBA in soils [65]. After introduction with strain HP-S-01 (1 × 10^6^ CFU/g), laboratory results showed that the removal rate of the initial doses of β-cypermethrin and 3-PBA (50 mg/kg) were higher in non-sterilized soils (87.8% and 79.3%, respectively) when compared to sterilized soils (80.5% and 73.1%, respectively). This indicated that soil microorganisms may have enhanced the ability of strain HP-S-01 in removing β-cypermethrin and 3-PBA. Enhanced removal may be due to the synergistic abilities between strain HP-S-01 and the indigenous soil microbial community in degrading β-cypermethrin and 3-PBA. Similar results were observed in field experiments whereby strain HP-S-01 had removed 81.1% and 73.5% of initial concentrations of β-cypermethrin and 3-PBA, respectively from the soil after 10 days [65].

Lindane or γ-hexachlorocyclohexane (γ-HCH) is a toxic insecticide that has been extensively used for agriculture. Lindane residues can leak through soil surfaces to ground water, resulting pollution of aquatic ecosystems [78]. Saez et al. (2014) demonstrated efficient lindane removal from concentrated soil slurry by a *Streptomyces* consortium (consisting of *Streptomyces* strains A2, A5, A11, M7) immobilized in cloth sachets [79]. From testing various inoculum sizes, lindane, and slurry concentration, best lindane removal (35 mg/kg of soil) from concentrated slurry (soil: water ratio of 2:3) was achieved within 7 days of incubation using *Streptomyces* inoculum of 1 × 10^7^ CFU/g. As for diluted slurry (soil: water ratio of 1:4), 28.7 mg/kg of soil of lindane was removed by the immobilized *Streptomyces* consortium after 14 days [79]. Later, Saez et al. (2018) investigated the bioaugmentation effect of a mixed culture consisting of the *Streptomyces* consortium (strains A2, A5, A11, M7) and fungi, on their capacity to remove lindane (100 mg/kg) from biomixture samples formulated with clayey soil, sandy soil, and silty loam soil [80]. It was observed that at 60 days of incubation, lindane removal achieved by the bioaugmented biomixtures were about 81–87% while the non-bioaugmented mixtures only showed 55–70% of lindane removal [80].

Moreover, Raimondo et al. (2020a) reported microcosm studies involving the applications of bioaugmentation with *Streptomyces* consortium (*Streptomyces* strains A2, A5, A11, M7) and biostimulation with sugarcane filter cake to bioremediate lindane-contaminated soils [81]. Inoculation with the *Streptomyces* consortium (2 g/ kg, wet weight) and biostimulation resulted in lindane (2 mg/kg) removal of 70.8%, 61.4% and 86.3% from clayey soil, silty loam soil and sandy soil, respectively after 14 days. In contrast, non-bioaugmented and non-biostimulated mesocosms only showed about 26–35% of lindane removal [81]. Later, in another study, Raimondo et al. (2020b) reported successful scaling-up from microcosms to mesocosms for the combined applications of bioaugmentation with *Streptomyces* consortium and biostimulation with sugarcane filter cake for the bioremediation of lindane-contaminated soils [82]. After 63 days of incubation, inoculation with the *Streptomyces* consortium (2 g/ kg, wet weight) and biostimulation showed lindane (2 mg/kg) removal of 68.6%, 80% and 82.6% from clayey soil, silty loam soil and sandy soil, respectively. For non-bioaugmented and non-biostimulated mesocosms, only about 32–38% of lindane removal observed [82].

## 6 Actinobacteria with heavy metal resistance and capabilities in heavy metal removal

Heavy metals are metallic elements found in natural sources (soil, sediments, water, air and living organisms), and also from anthropogenic sources. Heavy metals of anthropogenic origin generate a continuous and everlasting pollution and are usually related to agriculture, industrial and urbanization sectors [2,83]. Actinobacteria have been found to be either the second or third major groups in contaminated soils [25], [26], [27] and therefore, is a potential group that can be used for cleaning heavy metal pollution together with the indigenous microorganisms in soil. Besides that, major probiotics such as Bifidobacteria, found in human intestinal tract have also been proven to be able to remove heavy metals, especially from water samples [84,85]. Over the years, several *Streptomyces* and non-*Streptomyces* actinomycetes of the genus *Acinetobacter, Arthrobacter, Bifidobacterium, Micrococcus, Nocardia, Nocardiopsis, Propionibacterium* and *Rhodococcus* have been identified to exhibit heavy metal resistance, with some having capabilities in heavy metal removal (Table 3), which indicates their potential capacity as tools for heavy metal bioremediation.

**Table 3:** Actinobacteria that exhibits heavy metal resistance and with capabilities in heavy metal removal.

Actinobacteria	Heavy metal resistance	Additional info	Ref
Actinobacteria TY046–21, Actinobacteria TY046–078, Actinobacteria TY046–017,	Arsenic, lead, nickel, mercuric ion	▪ Isolated from tin tailings and forest soil ▪ Strains TY046–21 and TY046–078 show multi-metal tolerance to arsenic, lead and nickel ▪ Only strain TY046–017 show tolerance to mercuric ion	[86]
*Acinetobacter haemolyticus*	Chromium	▪ Isolated from effluent of textile-related ▪ Resistance to Cr(VI) < Zn < Cd < As(III) < Pb based on LC_50_ ▪ Removal of Cr(VI) contamination in water system	[87]
*Arthrobacter ramosus* G2	Cadmium, cobalt, zinc, chromium, mercury	▪ Isolated from mercuric salt-contaminated soil ▪ Able to bioaccumulate cadmium, cobalt, zinc, chromium, and mercury, and detoxify redox-active metals (chromium, mercury)	[88]
*Bifidobacterium angulatum*	Cadmium, lead, arsenic	▪ Effective removal of cadmium, lead and arsenic in artificially contaminated water	[89]
*Bifidobacterium longum* 46, *Bifidobacterium lactis* Bb12	Cadmium, lead	▪ Able to bind and remove cadmium and lead efficiently and rapidly in water	[84]
*Bifidobacterium breve Bbi99/E8*, *Propionibacterium freudenreichii shermanii JS*	Cadmium, lead	▪ Efficient cadmium and lead removal	[85]
*Micrococcus* sp. AL06Ni and AL94Co	Nickel, cobalt	▪ Isolated from a heavy metal contaminated soil	[90]
*Nocardia* sp. CA114Cr	Chromium, nickel	▪ Isolated from a heavy metal contaminated soil ▪ Able to tolerate 15 mM nickel	[90]
*Nocardia* strain B21	Iron	▪ Removal of 95.5% of iron in wastewater	[91]
*Nocardia* sp. MORSY2014	Chromium, zinc, nickel	▪ Isolated from polluted wastewater from industrial regions ▪ Removal of chromium (59.4%), zinc (62.8%), and nickel (50.5%) by isolate biomass	[92]
*Nocardiopsis* sp. MORSY1948	Zinc, nickel, chromium	▪ Isolated from polluted wastewater from industrial regions ▪ Removal of zinc (67.4%), nickel (60.1%), and chromium (47.4%) by isolate biomass	[92]
*Nocardiopsis* strain M13	Lead	▪ Removal of 56.5% of lead on wastewater	[91]
*Rhodococcus* sp.	Cadmium, lead, aluminum, iron	▪ Isolated from landfill-leachate contaminated soil	[93]
*Rhodococcus* sp. CA60Co, AL05Ni, AL32Cd and AL03Ni	Cadmium, chromium, nickel	▪ Isolated from a heavy metal contaminated soil	[90]
*Streptomyces* sp. F4	Cadmium	▪ Isolated from polluted soil of a former uranium mine ▪ Able to absorb and complex cadmium	[94]
*Streptomyces* sp*.* M46	Chromium	▪ Isolated from copper filter plant ▪ 50% removal of chromium after 3 days incubation	[95]
*Streptomyces* sp. strain C11	Copper, iron, manganese	▪ Removal of 93% of 0.4 mg/L iron, 90.9% of 0.03 mg/L manganese and 77.5% of 0.027 mg/L in wastewater	[91]
*Streptomyces* sp. strain FM1 and FM2	Lead	▪ Isolated from soil of industrial wastewater treatment plant ▪ Able to grow at ≥5000 mg/l	[96]
*Streptomyces acidiscabies* E13	Nickel	▪ Isolated from polluted soil of a former uranium mine ▪ Produce nickel struvite	[97, 98]
*Streptomyces griseus* NCIM 2020	Chromate	▪ Completely reduced 25 mg/L of Cr(VI) to Cr(III) within 24 hours	[99]
*Streptomyces rochei* ANH	Chromium, cadmium, lead	▪ Isolated from seacoast sediment in Alexandria, Egypt ▪ Removal of toxic heavy metals (nickel, copper, lead, cadmium, and chromium) in industrial wastewater ▪ Efficient removal of phytotoxin in tannery wastewater	[100]
*Streptomyces matansis* BG5, *Streptomyces vinaceus* CRF2, *Streptomyces* sp. CRF14, *Streptomyces* pulcher CRF17	Chromium	▪ Isolated from saline farmlands of Punjab, Pakistan ▪ Resistant to chromium at 1000 mg/L ▪ Strain BG5, CRF2 and CRF14 were able to reduce chromium completely at 150 mg/L after 168 h	[101]
*Streptomyces thermocarboxydus*	Chromium	▪ Isolated from estuarine sediment in Tokyo Bay ▪ Resistant to a concentration of 150 mg/l of chromium ▪ Reduced 65.4% of Cr(VI) after 4 days	[102]
*Streptomyces zinciresistens*	Zinc, cadmium	▪ Isolated from zinc-copper mine ▪ Resistant to 35 mmol/L zinc and 22 mmol/L cadmium	[103]

## 7 Bioaugmentation of heavy metals by actinobacteria

The success of bioaugmentation depends on the survival of the inoculated strains upon introduction to the bioaugmented soil. Hence, in heavy metal contaminated sites, actinobacteria with capabilities to resist or tolerate high concentrations of heavy metals will have better adaptability and survival in these stressed environments, making them suitable to be applied for heavy metal bioremediation. Although various studies have identified heavy metal resistant actinobacteria, and with some showing capabilities in heavy metal removal (Table 3), the potentials of heavy metal resistant actinobacteria for use in bioaugmentation approach have not been much explored. Table 4 lists some actinobacteria that have been studied for heavy metal removal via bioaugmentation.

**Table 4:** Studies on bioaugmentation of heavy metals using actinobacteria.

Actinobacteria	Heavy metal	Additional info	Ref
*Amycolatopsis tucumanensis* DSM 45,259	Copper	Copper removal up to 31% in loamy soil samples	[107]
*Rhodococcus* sp	Lead, copper, aluminum	Significant reduction of heavy metals in soil after 100 days treatment	[108]
*Streptomyces* sp. R25	Cadmium	Cadmium reduction in soil after 3 weeks of incubation	[111]
*Streptomyces* sp. MC1	Chromium	94% reduction of chromium in soil samples after 7 days	[112]
*Streptomyces* strains (M7, MC1, A5) and *Amycolatopsis tucumanensis* DSM 45,259	Chromium	▪ For single culture: ▪ Strain MC1 removed 94% of chromium in liquid system ▪ Strain M7 removed 50% of chromium in soil sample ▪ For mixed culture: ▪ Removal of >90% of chromium in liquid system and 64.4% of bioavailable chromium in soil	[113]
*Streptomyces* strains (M7, MC1, A5) and *Amycolatopsis tucumanensis* DSM 45,259	Chromium	Chromium removal until not detectable amounts by the consortium after 14 days of incubation	[114]

*Amycolatopsis* sp. AB0 or *Amycolatopsis tucumanensis* DSM 45,259, isolated from copper polluted sediments [104,105], demonstrated remarkable copper resistance and was able to remove copper up to 70% in minimal media [106]. Further study on bioaugmentation of copper in loamy soil samples showed that strain DSM 45,259 was able to reduce copper up to 31%. Strain DSM 45,259 was able also to colonize well in the copper contaminated soil microcosms, which confirmed its resistance towards copper in the soil [107].

Emenike et al. (2017) reported on the isolation of nine bacteria from landfill-leachate contaminated soil including *Rhodococcus* sp. in Malaysia [108]. In this study, the residual concentrations of lead, copper and aluminum in the soil were significantly reduced after 100 days of treatment by two different consortia, whereby one consortium consisted of all isolated bacteria (LSA) while another consortium consisted of *Bacillus* sp., *Rhodococcus* sp. and *Lysinibacillus* sp. (LSB). The LSB consortium consisting of *Rhodococcus* sp. was able to reduce high amounts of heavy metal from the leachate contaminated soil, indicating that *Rhodococcus* sp. is a potential candidate in the combination of bacteria for bioaugmentation that can speed up the removal of heavy metal processes [108].

In a bioaugmentation study done by Strachel et al. (2020), two out of four strains isolated from loamy sand contaminated with zinc were non-*Streptomyces* species, *Gordonia amicalis* KM113029.1 and *Leifsonia* sp. KJ191763.1 [109]. It was observed that soil inoculation with the zinc-resistant microbial consortium was able to minimize the adverse effects of zinc contamination in soil. However, unlike other bioaugmentation studies, the microbial consortium in this study was unable to reduce the bioavailable zinc in the loamy sand sample [109].

*Streptomyces* sp. R25 was isolated from river sediments which were contaminated with heavy metals [110]. Jézéquel and Lebeau (2008) studied strain R25 for the bioaugmentation of cadmium and concluded that strain R25 was competitive towards indigenous microorganisms and can colonize in the soil [111]. Interestingly, strain R25 was also able to reduce cadmium after 3 weeks of incubation [111].

In another study, Polti et al. (2007) successfully isolated *Streptomyces* sp. strain MC1 from sugarcane and demonstrated its ability to reduce Cr(VI) in liquid minimal medium [95]. Later, Polti et al. (2009) investigated the ability of strain MC1 to reduce Cr(VI) [112]. Strain MC1 was proven to be able to reduce 50 mg/kg of Cr(VI) almost completely (94%) in the normal condition of soil samples after 7 days without any previous treatment and no additional of substrate onto the soil samples. Moreover, the reduction of Cr(VI) activity by strain MC1 was not affected by the indigenous microbial flora in soil samples [112].

Apairicio et al. (2018a) studied three different *Streptomyces* strains (*Streptomyces* sp. M7, MC1, A5) and one non-Streptomyces strain, *Amycolatopsis tucumanensis* DSM 45,259, isolated from different soils and sediments contaminated with heavy metals and organochloride pesticides, and investigated the ability of pure and mixed cultures to remove chromium in minimal media as well as in soil artificially polluted with chromium [113]. Overall, the removal of bioavailable chromium from soil by actinobacteria was found to be lower than in liquid system. Using single cultures, strain MC1 showed the maximal removal of chromium in the liquid system up to 94% while in the soil sample, strain M7 was able to remove only 50% of the bioavailable fraction of the metal. In the experiment using mixed cultures, the greatest chromium removal efficiency in liquid was obtained in the consortium consisting all four strains, whereby >90% of chromium was removed in the liquid system while 64.4% of bioavailable chromium was removed in the soil [113].

In another study, Apairicio et al. (2018b) used the same actinobacteria consortium to remove chromium and lindane in environmental co-contaminated soils [114]. The bioaugmentation experiment in six different soils collected showed that the consortium was able to reduce the concentration of chromium until not detectable amounts, in five soil samples after 14 days of incubation at optimal temperature and moisture conditions. Chromium removal from one soil sample was not successful due to the inability of the actinobacteria consortium to grow in the alkaline soil condition (pH 9.72). Furthermore, lindane concentrations in the co-contaminated soil studied showed reduction after inoculations with the consortium, indicating the potential of the four strains consortium as a promising tool for the bioremediation of soils co-contaminated with heavy metals and pesticides [114].

## 8 Conclusion and future prospects

Due to the continuous development on industrial activities, the spread of contaminants worldwide is unceasing as there are insufficient monitoring on the negative effects of these contaminants on the environment. This had led to various research for methods that can improve the conditions of these polluted environments. As such, bioaugmentation with actinobacteria is a beneficial environmentally friendly approach to help restore the environment. Actinobacteria have been proven to be abundant in contaminated soils and are able to metabolize contaminants for their growth. Furthermore, the potentials of actinobacteria in degrading pesticides and removing heavy metals have been demonstrated by various studies, indicating their potential capacities as tools for contaminant bioaugmentation.

However, the bioaugmentation strategy remains a challenging method for efficient elimination of contaminants as there are still limited information on the inoculated strains introduced into the bioaugmented soil. Bioaugmentation efficiency depends mostly on the degrading activity of the inoculated microorganisms and their ability to survive with indigenous microorganisms. Yet, death of the inoculated strains have been observed after introduction to a site, due to abiotic and biotic stresses, and distribution of the introduced strains were limited in the soil matrix [9,18]. Hence, new strategies are needed to improve the efficiency of bioaugmentation. A possible strategy is through quorum sensing modulation to stimulate biofilm formation of the inoculated and soil indigenous microorganisms. Quorum sensing can be controlled and regulated by adding auto-inductors or inhibitors, which may influence biofilm formation and colonization of microorganisms and their bioaugmentation effect [9,40]. Moreover, molecular techniques such as quantitative PCR, e-GFP-tagging, RT-qPCR and stable isotope probing, can be used to monitor the survival and activity of the inoculated strains introduced into the bioaugmented soil [115]. This will be useful for evaluating the performance of the inoculated strains under *in situ* conditions and will be helpful to improve bioaugmentation effectiveness.

## CRediT authorship contribution statement

**Christina-Injan Mawang:** Conceptualization, Writing – original draft, Writing – review & editing, Validation, Visualization. **Adzzie-Shazleen Azman:** Conceptualization, Writing – original draft, Writing – review & editing, Validation, Visualization. **Aalina-Sakiinah Mohd Fuad:** Writing – review & editing. **Mariana Ahamad:** Writing – review & editing, Validation.

## Declaration of Competing Interest

The authors declare no conflict of interest.
